# Supplementary material for: Reference Sequence Browser: An R application with a user-friendly GUI to rapidly query sequence databases
Source: PLoS One. 2024 Oct 31;19(10):e0309707. doi: 10.1371/journal.pone.0309707 (PMC11527216; doi:10.1371/journal.pone.0309707)
Supplement: Attachment — Submitted filename: Response_to_Reviewers.pdf [file pone.0309707.s002.pdf]

Dear editors and reviewers,

Thank you very much for your time and constructive feedback for our manuscript. All the comments were very useful and we have revised and improved the manuscript accordingly.

Below are the reviewer's and academic editor's comments (in regular black font), and below each comment is our response (in light blue font).

1. Please ensure that your manuscript meets PLOS ONE's style requirements, including those for file naming. The PLOS ONE style templates can be found at https://journals.plos.org/plosone/s/file?id=wjVg/PLOSOne_formatting_sample_main_body.pdf and https://journals.plos.org/plosone/s/file?id=ba62/PLOSOne_formatting_sample_title_authors_affiliations.pdf

Some of the style requirements mentioned above do not apply to the LaTeX requirements (such as the symbol legend for which the LaTeX and the word requirement differs), at least that's what we were told by PLOS however, we have, made a couple of adjustments as we did have a couple of minor issues:

- We have updated the author affiliations so they match the requirements
- Added initials to the corresponding author after the email

2. Please assign your protocol a protocols.io DOI, if you have not already done so, and include the following line in the Materials and Methods section of your manuscript: "The protocol described in this peer-reviewed article is published on protocols.io (https://dx.doi.org/10.17504/protocols.io.[...]) and is included for printing purposes as S1 File." You should also supply the DOI in the Protocols.io DOI field of the submission form when you submit your revision.

We assigned a DOI to the protocol and have added the above mentioned line together with Protocol DOI.

3. We note you have included a table to which you do not refer in the text of your manuscript. Please ensure that you refer to Table 1 in your text; if accepted, production will need this reference to link the reader to the Table.

We have added a reference to Table 1 in the text.

4. Please include captions for your Supporting Information files at the end of your manuscript, and update any in-text citations to match accordingly. Please see our Supporting Information guidelines for more information: http://journals.plos.org/plosone/s/supporting-information.

We have added a caption for our Protocol as that counts as supporting information.

5. Please review your reference list to ensure that it is complete and correct. If you have cited papers that have been retracted, please include the rationale for doing so in the manuscript text, or remove these references and replace them with relevant current references. Any changes to the reference list should be mentioned in the rebuttal letter that accompanies your revised manuscript. If you need to cite a retracted article, indicate the article's retracted status in the References list and also include a citation and full reference for the retraction notice.

We have included several new references in our paper specifically for the newly created Related Works section. Other than these additions, there have been no changes made, whether in terms of removing or adding other works.

2. Has the protocol been described in sufficient detail?

Due to the reviewer's feedback, we decided to go back to the protocol and improve it a little more. The instruction did not ask for a marked up version of the protocol but we are listing the few changes made below:

1. Added a new figure in step 20.1
2. Added two new figures in step 20.2
3. Added a couple small paragraphs clarifying steps 20.1-20.2 further.
4. Made some small grammatical changes here and there.
5. Added further clarification on the NCBI Key and how to obtain one
6. Added clarification on BOLD barcodes not being chosen in Step 20.
7. Added clarification on CRUX barcodes not being chosen in Step 23.

Reviewer #1:

1. There are some issues that could be improved. One is to review potentially related/similar work (e.g. crabs: Jeunen, G.-J., Dowle, E., Edgecombe, J., von Ammon, U., Gemmell, N. J., & Cross, H. (2023). CRABS—A software program to generate curated reference databases for metabarcoding sequencing data. Molecular Ecology Resources, 23, 725–738. https://doi.org/10.1111/1755-0998.13741
taxalogue: Noll NW, Scherber C, Schäffler L. taxalogue: a toolkit to create comprehensive CO1 reference databases. PeerJ. 2023 Dec 4;11:e16253. doi: 10.7717/peerj.16253. PMID: 38077427; PMCID: PMC10702336.) noting overlaps and differences and the degree to which the work fills a gap.

Thank you for bringing this up, this is a very good point. The main reason we didn't write one originally is because we haven't seen a Related Works section in many other eDNA tool articles. That said, our primary goal is to lower the barrier of entry for new eDNA scientists to jump into the field, and we believe including a Related Works section would help our target audience. Therefore, we have followed your recommendation and added a related works section addressing other bioinformatic tools out there, noting the differences and the importance of the RSB.

2. There are also some sections that could use some clarifications and corrections. In the section Case 1.1 under the third paragraph, the text states, 'To get more information, the user would then

inspect the CM like the Fig. 3.' Figure 3 appears as a summary table rather than a coverage matrix (CM) as stated.

Yes, that was a mistake when uploading the figures, fig2 was supposed to be fig3 and fig3 should have been fig2. Thank you for catching this. We have now addressed this and all the figures should now be ordered correctly. Unfortunately, we imagine this would have made the text for use case 1.1 confusing as it talks about interpreting these tables. We apologize for the inconvenience, in addition to fixing the figures we have also made a few changes in the text to make it clearer how one can interpret the tables.

3. A little more careful alignment between text and figures would alleviate some potential confusions. For example, the text says, 'As shown in the summary table, of the 11 invasive species searched, six organisms had at least one sequence.' It is not clear how this information was derived from the summary table since it is not organized by organism. The text further says, 'The vast majority of the sequences were found in the CO1 barcode and includes five organisms but the 16S barcode captures all six detected 148 organisms.' That the majority are found in the CO1 barcode is clear from the summary table but one needs to refer to the coverage matrix (CM) to determine that there were 5 organisms detected under CO1 and six detected under 16S. Similar additional clarification would help with the next paragraph. The text states, 'one can see examples of the three different ways in which the RSB highlights missing information in the CRUX databases. Firstly, Lithobates catesbeianus, Dreissena polymorpha, and Euwallacea kuroshio are not in the database. It would help to point out to readers the pattern that depicts this. From the Figure 2 example one can see that there are no sequences indicated for Lithobates catesbeianus (no numbers other than zero), but the text family and phylum appear so this makes sense. Dreissena polymorpha does have sequences appearing for 18S and 16s so the above statement that there are no sequences for this organism appears incorrect. Perhaps this was meant to be Dreissena bugensis which does show no sequences appearing across the set of barcodes?

We will address these two comments together as they both refer to Use Case 1. We believe that the misordering of the figures made this harder to understand. However, we have also revised and improved two main things.

The figures themselves, for which we have added red bounding boxes to better highlight what we are looking at. In the hopes that by making it clear what we are focusing on it is easier to follow the text.

We have also improved the clarity and explanation of much of the text. In particular, we have changed the paragraphs explaining the Summary Data and Coverage Matrix, and added an explanation for why we chose to focus on the CO1 barcode.

That said, to directly address some of your concerns, below we leave some additional clarification:

1. The rows in the Summary Table correspond to barcodes, so we can look at each row and determine how many reference sequences each barcode has. In addition, the column titled "Organisms with at least one sequence" lets us know that 6 organisms in 16S have at least 1 reference sequence. Similarly, the CO1 barcode has 5 organisms with at least 1 sequence. Therefore, we don't need to look at the CM to determine how many organisms are actually covered by each barcode.
2. The reason we mentioned that those species had no sequences was because we were only looking at the CO1 Barcode/Column only to limit the scope of the discussion. Our wording was a bit confusing because when we mentioned the word "database" later we were referring to the CRUX CO1 database (This has now been made much clearer). We meant to say that these 3 organisms were not covered by CO1, but sequences exist for other species in the genera Lithobates, Dreissena, and Euwallacea. Also, we have added Dreissena Polymorpha as we did not mention originally even though it has results at the genus level (this was fixed in the new revised manuscript as well).

We hope that the fixed ordering of the figures and the rewording of Use Case 1.1 explaining things in a little more detail and with clearer explanations addresses all the concerns mentioned above.

4. With respect to Figure 4. The text might be amended from this: 'Here, there are CO1 sequences for species that were not present in CRUX. To 'The NCBI search results show that there are CO1 sequences for species (Lithobates catesbeianus, Dreissena polymorpha, Dreissena bugensis and Euwallacea Kuroshio) that were not present in CRUX.

We have made the change as we agree this will make things clearer.

5. With respect to Figure 5, the caption is,' Example of NCBI Summary Data table for the genus Xenopus' However the figure title is 'BOLD coverage matrix'. It appears that Figures 5 and 6 are inverted with respect to the captions /associated png files.

Yes, when uploading the figures to PLOS, as they must be separated from the paper they were inverted. This has now been fixed.

6. In summary the work has benefit for supporting search for and comparison of sequence availability across a set of data repositories. The authors however could provide some further discussion of related work (as pertinent) and provide more specifics and clarifications to assist readers in interpreting the tabular results.

We believe we have addressed everything and hope the text is more clear as interpreting the results is a key component of our tool.

Reviewer #2:

1. Line 16: BOLD stands for Barcode of Life Data System; authors should make sure to use its correct name.

Thank you for catching this, it has been fixed.

2. Lines 35-38: Example for the UC system is interesting but difficult to put into context. Is this expected to be high or low in comparison to other schools? Average? I realize that this is merely to illustrate that few students may have opportunities to gain experience in programming, but I wonder if the high-powered UC system actually represents an overestimate of how many students are exposed to this training. Maybe something like that could be mentioned.

This is something that we hadn't considered. **However, we decided to keep this as is** because while this could be an overestimate, the study covers both the CSU and UC school systems, and therefore includes a broad diversity of schools and students. We worry about the accuracy of such a claim without more data.

3. Line 131: This paragraph is useful; I just worry that an inexperienced reader could interpret its message to say that a barcode should be chosen based on how many organisms it covers when a barcode should probably be chosen based on numerous factors (certainly inclusive of coverage, but also factors like species-level genetic variability). Maybe authors could mention something like that users can evaluate this factor "in addition to other factors specific to their study."

Thank you; we have made a change accordingly in the paper to make sure this doesn't confuse anyone.

4. Figures 1-3, 6: I was curious why trnL is included in these tables when it is a cpDNA marker and would not be used in exploring barcodes of amphibians. Upon further inspection, it seems that in the RSB itself the "barcodes" rows/columns appearing in these figures are just the standard list included in outputs. Does this mean, then, that coverage of only one cpDNA barcode can be explored for plants? If so, it may be worth a more detailed discussion of the utility of this tool for various types of organisms, or stating that this is not an exhaustive list of markers and researchers might be interested to use others not in the RSB (e.g., trnL has become the new standard for plants but those interested in a multi-marker approach or who might find utility for a more widely used marker like rbcL might benefit from knowing that they have other

options). Since this tool is introduced as one that is beginner-friendly, additional information on why that set list of markers is used in the app and how individuals might consider alternatives (or expand on this framework to compare a standard barcode to lesser-used barcodes) could be helpful.

This may not have been clear in our paper, but in the CRUX pipeline, we search every single available database. Each column corresponds to a barcode (e.g., CO1, 16S, trnL), and each of those columns represents a database in CRUX. In other words, the user doesn't specify which barcodes to search for in CRUX; we simply search them all since there aren't that many. You are correct that trnL wouldn't be used for exploring barcodes for amphibians, and scientists would simply ignore that column if it's not relevant to their research. We explain this further in the protocol, but for clarification purposes, we have added more details in the paper to avoid any confusion.

5. Line 200: Creating local reference libraries for study systems of interest is crucial to high-resolution and reliable metabarcoding data. I wonder if their importance could be more explicitly stated in this paragraph (the development of local reference libraries is also synergistic with the goals of RSB including identifying taxonomic and geographic gaps in barcode data availability).

We have changed the wording to emphasize the importance of creating reference sequence databases.

6. Line 241: Could the authors briefly explain how users might deal with or anticipate homynyms in the framework of the RSB?

We have added a couple of options on how a user could go about dealing with Homonyms. Both options are using other tools as we, unfortunately, don't have a way to deal with them yet in the RSB.

In our protocol, we do talk about a feature that we have in the CRUX pipeline to alert a user about homonyms automatically. However, this feature is yet to be implemented in the other databases, and to avoid confusion, we have refrained from mentioning it in the paper.
